# Development of daily rhythmicity in heart rate and locomotor activity in the human fetus

**DOI:** 10.1186/1740-3391-3-5

**Published:** 2005-03-31

**Authors:** Paliko I Kintraia, Medea G Zarnadze, Nicolas P Kintraia, Ia G Kashakashvili

**Affiliations:** 1 K.V. Chachava Institute of Perinatalogy, Obstetrics and Gynecology, 38 Kostava Str., Tbilisi 0108, Republic of Georgia

## Abstract

**Background:**

Very little is known about the perinatal genesis of circadian rhythmicity in the human fetus. Some researchers have found evidence of rhythmicity early on in fetal development, whereas others have observed a slow development of rhythmicity during several years after birth.

**Method:**

Rhythms of fetal heartbeat and locomotor activity were studied in women with physiological course of pregnancy at 16 to 40 gestational weeks. Observations were conducted continuously for 24 h using the method of external electrocardiography, which provided simultaneous detection of the changes in maternal and fetal heartbeat as well as assessment of daily locomotor activity of the fetus. During the night-time, electroencephalogram, myogram, oculogram and respiration of the mother were registered in parallel with fetal external electrocardiography.

**Results:**

Although we found no significant daily rhythmicity in heart rate per se in the human fetus, we developed a new method for the assessment of 24-h fetal cardiotachogram that allowed us to identify daily rhythmicity in the short-term pattern of heart beating. We found that daily rhythmicity of fetal electrocardiogram resembles that of the mother; however, the phase of the rhythm is opposite to that of the mother. "Active" (from 9 a.m. to 2 p.m. and from 7 p.m. to 4 a.m.) and "quiet" (from 4 a.m. to 9 a.m. and from 2 p.m. to 7 p.m.) periods of activity were identified.

**Conclusion:**

A healthy fetus at gestational age of 16 to 20 weeks reveals pronounced rhythms of activity and locomotion. Absence of distinct rhythmicity within the term of 20 to 24 weeks points to developmental retardation. The "Z"-type fetal reaction, recorded during the "quiet" hours, does not indicate unsatisfactory state, but rather is suggestive of definite reduction of functional levels of the fetal physiological systems necessary for vital activity.

## Background

Animal homeostasis is based on rhythmic activity of the physiological systems of an organism, whereas these rhythmic processes bring the physiological functions into line with environmental rhythmicity. Studies that highlight the regulating role of the suprachiasmatic nucleus in the course of circadian rhythmic activity [15,21,27], that confirm the significance of melatonin in the development of sleep-wakefulness rhythms [12,20,24,26], and that underscore the importance of cryptochromal proteins in the generation of daily rhythmic activity [11,25,28] have provided profound insights into the rhythmic mechanisms of human physiological functions. However, much remains to be learned. Among the challenging questions is that concerning the perinatal genesis of circadian rhythmicity of the human fetus. Few investigations have been conducted in this area. Some researchers have found evidence of rhythmicity early on in fetal development, whereas others have observed a slow development of rhythmicity during several years after birth [19,22,23].

The Research Institute of Perinatal Medicine, Obstetrics and Gynecology has been conducting investigations on fetal rhythmicity since 1978 with three major goals:

1. Determination of rhythmicity of the heartbeat and locomotor activity of the human fetus, including the interdependence between maternal and fetal biological rhythms (Research works 1978–1979);

2. Differentiation of the characteristics of the human fetus response to exertion (workload) tests with regard to natural changes in the functional state of the fetus related to the run of its biological clock (Research works 1980–1986); and

3. Determination of the time of onset and formation of fetal biological rhythms (Research works 1989–1993).

## Method

### Stages 1 and 3

#### Subjects

The subjects in Stage 1 were 18 volunteer pregnant women, aged 19 to 26 years, at the gestational period of 36 to 40 weeks. They were informed of the format and extent of the trials beforehand. The subjects in Stage 3 were 28 pregnant women examined at the gestational period of 16 to 28 weeks (16 to 17 weeks: n = 1; 17 to 18 weeks: n = 3; 18 to 19 weeks: n = 2; 19 to 20 weeks: n= 2; 20 to 21 weeks: n = 3; 21 to 22 weeks: n = 2; 22 to 23 weeks: n = 4; 23 to 24 weeks: n = 3; 24 to 28 weeks: n = 8).

Catamnestic data for the 46 pregnant women observed showed that in all cases newborns were healthy, being evaluated by Apgar Scale as 8–9 points (19 cases) and 9–10 points (27 cases).

#### Procedure

Recording sessions were conducted in electrically isolated rooms following 2 or 3 days of preliminary adaptation. Routine living conditions were maintained as much as possible. The participants were thoroughly screened and selected for physiological pregnancy. The observation was performed uninterruptedly during 24 hours, using the method of external electrocardiography (ECG) to identify maternal and fetal heart beat as well as fetal locomotor activity. External ECGs of the fetus and EEGs of the mother were recorded by means of 16 channel electroencephalograph " MEDICOR ", Hungary. Paper-tape velocity was 30 mm/sec.

The recordings obtained were processed by the calculation of maternal and fetal heart rate (FHR) in 5-minute intervals and transferred onto the cardiotachogram representing a graph of intra-minute fluctuations of HR. Based on 24-hour uninterrupted recording of fetal external ECG, a 24-hour cardiotachogram was plotted for every hour (from 1 to 2, 2 to 3, 3 to 4 etc.). The analysis of hourly cardiotachogram was done according to the following parameters: hourly fluctuations in maternal and fetal HR, intra-minute fluctuation of HR, and baseline rhythm. The baseline was defined as the level obtained at 7 a.m., under the conditions of basic metabolic rate. The values of the parameters under study at 12 p.m., 5 p.m., 10 p.m., and 2 a.m. were compared to the levels at 7 a.m. (39, 40). Fetal movements were fixed at oscillation of isoelectrical line of the external ECG of the fetus, calculated by hour and a graph of hourly fetal movements was plotted. At night-time, external ECGs of the fetus and EEGs of the mother were recorded simultaneously with the above procedures. The EEGs were subjected to visual analysis, which consisted of the evaluation of wave duration, sleep amplitude, shape and general structure, sleep cycles, and phasic composition and duration.

### Stage 2

A total of 2,500 fetuses from carefully selected pregnant women with physiological course of pregnancy at the gestational term of 32 to 40 weeks were studied in Stage 2. Single-time recording of external ECG or external cardiotachogram (CTG) was conducted during 20–25 min. To determine the intrauterine condition of the fetus, functional tests were carried out 10–12 min following the commencement of the background recording. Breath-hold at expiration, physical exertion, sound stimulation, and non-stress test (NST) were used as functional loads. Breath-hold at expiration was carried out during 20–25 sec. For "sound load", a telephone handset was placed anteriorly on the abdominal wall in the fetal head projection area and a permanent sound was delivered at the frequency of 500–1000 Hz for 60 sec at an intensity of 105 decibel. The mothers wore ear-plugs during the stimulation. Physical exertion was given in the form of bending and straightening of the trunk 10 times for 45–50 sec. Functional state of the fetus was evaluated based on the analysis of a one-time CTG (focus being placed on the HR fluctuations, baseline rhythm, intra-minute oscillation, fetal reactivity to functional loads, and number of variable accelerations and decelerations). The trials were dynamically conducted during both "active" (9 a.m. to 2 p.m.) and "quiet" (2 p.m. to 7 p.m.) periods. The electrographic findings of the intrauterine fetus were correlated with evaluations by Apgar scale as well as with the data regarding the course of postnatal period. The time of the recordings was strictly fixed for each observation of the fetus. Differences between the parameters under study were considered reliable at p < 0.05.

## Results and Discussion

### Stage 1

The methodical approach utilized in our study of daily rhythms of external ECG allowed us to perform a digital analysis of hourly values of maternal and fetal heart rate during 24-hours of uninterrupted recording. We were able to evaluate changes in the functional levels of two processes in the fetus: heart action and locomotor activity. The uninterrupted recordings of fetal ECGs allowed us to use a new self-designed technique for analysis of daily cardiotachograms (CTG) of the fetus and the pregnant woman. The analysis of heart rate changes in healthy pregnant women showed that function enhances from 7 a.m. (66.5 bpm) to 12 p.m. (79.5 bpm), slightly reduces, and then continues to increase until 5 p.m. (76.5 bpm). Minimal heart rate is observed at 2 a.m. (61 bpm). Thus, in healthy pregnant women, the daily rhythms of HR fluctuations seem to be preserved (Table 1).

**Table 1:** Hourly layout for 24-hour period frequencies of maternal and fetal heart rate rhythms. (36 – 40 weeks gestation)

**Time of day (h)**	**Mother (bpm)**	**Fetus (bpm)**
7 – 8	66.5 ± 1.3 *	133 ± 1.9 **
8 – 9	68 ± 1.2 *	133 ± 2.1 **
9 – 10	68.5 ± 1.3 **	132 ± 2.0 **
10 – 11	72 ± 1.4 **	136 ± 2.1 **
11 – 12	76 ± 1.4 **	135 ± 2.3 **
12 – 13	79 ± 1.3 *	136 ± 2.3 **
13 – 14	79 ± 1.2 *	134 ± 1.9 *
14 – 15	75 ± 1.2 *	136 ± 2.1 **
15 – 16	76 ± 1.1 *	133 ± 2.3 *
16 – 17	76 ± 1.1 *	132 ± 1.8 **
17 – 18	76.5 ± 1.2 *	133 ± 2.2 **
18 – 19	79 ± 1.0 *	135 ± 1.8 **
19 – 20	71 ± 1.1 *	131 ± 1.8 *
20 – 21	72 ± 1.1 **	133 ± 2.0 *
21 – 22	69 ± 1.2 *	135 ± 1.9 **
22 – 23	70 ± 1.1 *	134 ± 1.8 **
23 – 24	65 ± 1.0 *	136.5 ± 2.0 **
24 – 1	64 ± 1.2 *	138.5 ± 1.9 *
1 – 2	65 ± 1.1 *	138.5 ± 2.2 *
2 – 3	61 ± 1.2 *	133.5 ± 1.9 **
3 – 4	64 ± 1.1 *	136 ± 1.8 **
4 – 5	62 ± 1.1 *	132 ± 1.9 **
5 – 6	66.5 ± 1.1 *	133 ± 2.1 **
6 – 7	68 ± 1.0 *	134 ± 2.2 **

* p < 0.001 (compared to heart rate at 7 am).

** p > 0.05.

**Figure 1:**
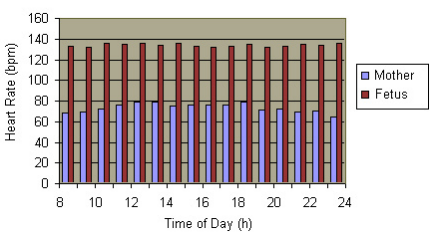
Hourly layout for 24-hour period frequencies of maternal and fetal heart rate rhythms (36 – 40 weeks gestation)

**Figure 2:**
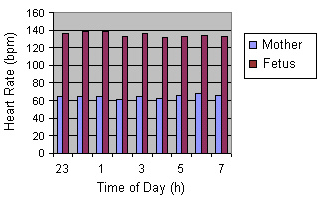
Hourly layout for 24-hour period frequencies of maternal and fetal heart rate rhythms (36 – 40 weeks gestation)

The study of 24-hour FHR fluctuations demonstrated that the HR magnitude at 7 a.m. was 133.0 ± 1.9 bpm; at 10 p.m. it was 136.5 ± 2.0 bpm, and at 2 a.m. the value approximated that at 7 a.m. (133.5 ± 1.6 bpm). Statistical processing of fetal HR magnitude during the 24-hour period did not show any significant difference between day and night measurements (Fig. 1, Fig. 2). Consequently, analysis of daily FHR fluctuations using the method of frequency changes computing did not permit to judge on the presence of daily periodicity of the fetal heart rhythm.

Karr et al [16] and Hellbrugge [13] investigated 24-hour heart rate rhythms of the mother and the fetus using single auscultations of maternal heart rate and pulse with 2-hour intervals throughout 24 hours. N.N. Konstantinova [10] and Hoppenbrowers [14] directed their investigations mainly toward the study of fetal heart rate changes during the mother's sleep. Our findings agree with Hellbrugge's [13] results indicating the occurrence of typical 24-hour HR rhythms in the pregnant woman. At the same time, FHR is more or less identical during the day time and at night, averaging 133.0 ± 5.0 bpm. Hoppenbrouwers [14] found that fetal HR did not undergo substantial changes during the sleep and waking periods of the mother. N.N. Konstantinova [10] reported a dependence of fetal HR on maternal sleep-wakefulness cycles.

**Figure 3:**
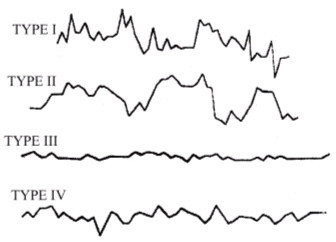
Types of cardiotachograms

We have undertaken the task of investigating the principles of conformity between maternal and fetal rhythms with the result of taking notice of heterogeneity in fetal cardiotachograms, which allowed us to classify four types of oscillations occurring on an hourly cardiotachogram in different combinations, and sequences, and continuously replacing one another. Depending on the differences in form, amplitude and duration, each of the oscilation types was designated by a proper term: "peak-like", "rounded", "flat" and "mixed" (Fig. 3). Type I ("peak-like") is characterized by rapid fluctuations of FHR during 5–10 sec with the intra-minute fluctuations of ± 12–30 bpm. Type II ("rounded") is characterized by a gradual intensification of fetal HR by ± 18–34 bpm and further gradual return to the initial values. Type III ("flat") is characterized by low intra-minute fluctuation of ± 1–4 bpm. Type IV ("mixed") is characterized by the baseline rhythm of 120–150 bpm and intra-minute fluctuation of ± 7–15 bpm.

**Figure 4:**
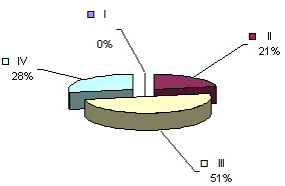
ECG of the fetus in "quiet" hours (4 a.m. to 9 a.m.) 36–40 weeks gestation

**Figure 5:**
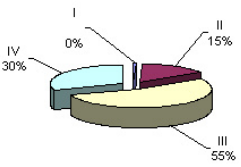
ECG of the fetus in "quiet" hours (2 p.m. to 7 p.m.) 36 – 40 weeks gestation

**Figure 6:**
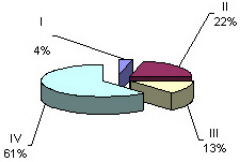
ECG activity of the fetus in "active" hours (9 a.m. to 2 p.m.) 36 – 40 weeks gestation.

**Figure 7:**
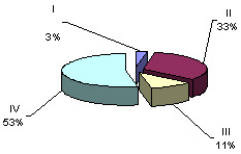
ECG activity of the fetus in "active" hours (from 7 p.m. to 4 a.m.) 36–40 weeks gestation

Having separated the 24-hour cardiotachograms into hourly parts, we carried out a careful visual and digital analysis of the findings and discovered certain irregularities in the distribution of one or another type of oscillations across a daily rhythmogram. Further, having computed the amount of time necessary for each type of cardiotachogram to repeat within one hour of the observation, we paid attention to the findings that the period when the background cardiotachogram is presented by "flat (III) type" oscillation (51% and 55 % of the recording time) was observed to occur twice during 24 hours, predominating over the other three types (Fig. 4, Fig. 5). The periods from 4 a.m. to 9 a.m. and from 2 p.m. to 7 p.m. were termed "quiet" hours. During the rest of the day, i.e. from 9 a.m. to 2 p.m., and from 7 p.m. to 4 a.m., the background cardiotachogram was represented by "mixed" oscillations with the prevalence of types I, II and IV (87% and 89% of the recording time). These periods were termed "active" hours (Table 2, Table 3, Table 4, Fig. 6, Fig. 7).

**Table 2:** ECG and locomotor activity of the fetus in "quiet" hours (4 a.m. to 9 a.m., 2 p.m. to 7 p.m.) (36 – 40 weeks gestation)

**Time (h)**	**Type**	**Duration**					**Time (h)**	**Duration**				
				
		**Oscillation type**		**Fetal movements**		**Recording**		**Oscillation type**		**Fetal movements**		**Recording**
				
		**Min.**	**Sec.**	**Min.**	**Sec.**	**Min.**		**Min.**	**Sec.**	**Min.**	**Sec.**	**Min.**
4–5	I	-	20 ± 0.2	-	-	54 ± 0.3	14–15	-	10 ± 0.2	-	-	40 ± 0.4
	II	16 ± 0.3	-	-	50 ± 0			3 ± 0.4	-	-	50 ± 0.1	
	III	20 ± 0.2	-	-	-			30 ± 0.4	-	-	-	
	IV	18 ± 0.2	-	-	-			7 ± 0.1	-	-	-	

5–6	I	-	-	1 ± 0.1	-	46 ± 0.2	15–16	1 ± 0.2	-	-	-	54 ± 0.3
	II	7 ± 0.4	-	-	-			7 ± 0.2	-	2 ± 0.1	-	
	III	30 ± 0.3	-	-	-			24 ± 0.1	-	-	-	
	IV	9 ± 0.3	-	-	-			21 ± 0.2	-	-	-	

6–7	I	-	30 ± 0.2	-	-	59 ± 0.2	16–17	-	20 ± 0.3	-	-	45 ± 0.1
	II	18 ± 0.1	-	1	30 ± 0.3			12 ± 0.1	-	1 ± 0.3	-	
	III	26 ± 0.1	-	-	-			21 ± 0.1	-	-	-	
	IV	15 ± 0.2		-	-			11 ± 0.1	-	-	-	

7–8	I	-	50 ± 0.1	-	-	50 ± 0.5	17–18	-	20 ± 0.1	-	-	45 ± 0.3
	II	12 ± 0.4	-	-	-			8 ± 0.2	-	1	50 ± 0.1	
	III	22 ± 0.4	-	1	5 ± 0.1			20 ± 0.1	-	-	-	
	IV	15 ± 0.3	-	-	-			16 ± 0.1	-	-	-	

8–9	I	-	20 ± 0.1	-	-	40 ± 0.2	18–19	-	10 ± 0.1	-	-	42 ± 0.1
	II	-	-	-	50 ± 0.1			4 ± 0.3	-	-	40 ± 0.2	
	III	28 ± 0.3	-	-	-			25 ± 0.2	-	-	-	
	IV	12 ± 0.3	-	-	-			12 ± 0.3	-	-	-	

**Table 3:** ECG and locomotor activity of the fetus in "active" hours (9 a.m. to 2 p.m.) (36 – 40 weeks gestation)

**Time (h)**	**Type**	Duration					**Time (h)**	Duration				
				
		**Oscillation type**		**Fetal movements**		**Recording**		**Oscillation type**		**Fetal movements**		**Recording**
				
		**Min**	**Sec**	**Min**	**Sec**	**Min**		**Min**	**Sec**	**Min**	**Sec**	**Min**
9–10	I	-	45 ± 0.2	7 ± 0.2	-	48 ± 0.2	12–13	-	10 ± 0.2	8 ± 0.1	-	30 ± 0.3
	II	6 ± 0.2	-	-	-			10 ± 0.1	-	-	50 ± 0.1	
	III	7 ± 0.3	-	-	-			2 ± 0.1	-	-	-	
	IV	35 ± 0.1	-	-	-			17 ± 0	-	-	-	

10–11	I	-	50 ± 0.4	3 ± 0.1	-	24 ± 0.3	13–14	2 ± 0.3	-	4 ± 0.3	-	48 ± 0.1
	II	8 ± 0.1	-	-	-			8 ± 0.1	-	-	-	
	III	3 ± 0.1	-	-	-			8 ± 0.2	-	-	-	
	IV	12 ± 0.01	50 ± 0.1	-	-			30 ± 0.1	-	-	-	

11–12	I	-	30 ± 0.2	4	50 ± 0.1	38 ± 0.2	Total from 9–14	6–42 ± 0.16	45 ± 0.26	26	50 ± 0.16	188 ± 0.22
	II	10 ± 0.3	-	-	-			25 ± 0.24				
	III	5 ± 0.3	-	-	-			116				
	IV	22 ± 0.2		-	-				50 ± 0.12			

**Table 4:** EEG and locomotor activity of the fetus in "active" hours (from 7 p.m. to 4 a.m.) (36 – 40 weeks gestation)

**Time (h)**	**Type**	Duration					**Time (h)**	**Duration**				
				
		**Oscillation type**		**Fetal movements**		**Recording**		**Oscillation type**		**Fetal movements**		**Recording**
				
		**Min**	**Sec.**	**Min**	**Sec.**	**Min**		**Min**	**Sec.**	**Min**	**Sec.**	**Min**
19–20	I	3 ± 0.1	-	4	50 ± 0	54 ± 0.1	24–1	2 ± 0.2	-	4 ± 0.1	-	53 ± 0.2
	II	15 ± 0.2	-	-	-			14 ± 0.1	-	-	-	
	III	4 ± 0.1	-	-	-			4 ± 0.4	-	-	-	
	IV	32 ± 0.2	-	-	-			33 ± 0.4	-	-	-	

20–21	I	-	10 ± 0.2	4 ± 0.2	-	30 ± 0.2	1 – 2	1 ± 0.1	-	6 ± 0.3	-	34 ± 0.4
	II	10 ± 0.1	-	-	-			11 ± 0.2	-	-	-	
	III	5 ± 0.1	-	-	-			7 ± 0.4	-	-	-	
	IV	15 ± 0.2	-	-	-			15 ± 0.2	-	-	-	

21–22	I	-	-	3 ± 0.1	-	39 ± 0.3	2 – 3	1 ± 0.2	-	4 ± 0.2	-	53 ± 0.1
	II	12 ± 0.4	-	-	-			18 ± 0.1	-	-	-	
	III	22 ± 0.4	-	-	-			7 ± 0.1	-	-	-	
	IV	15 ± 0.3	-	-	-			27 ± 0.2	-	-	-	

22–23	I	I	40 ± 0.2	16 ± 0.2	-	26 ± 0.3	3 – 4	-	30 ± 0.3	3	30 ± 0.2	31 ± 0.2
	II	7 ± 0.1	-	-	-			9 ± 0.1	-	-	-	
	III	-	-	-	-			7 ± 0.1	-	-	-	
	IV	18 ± 0.3	-	-	-			15 ± 0.3	-	-	-	

23–24	I	1 ± 0.3	-	3 ± 0.2	-	58 ± 0.1	Total from 19-4	12	20 ± 0.21	43	20 ± 0.16	388 ± 0.21
	II	23 ± 0.3	-	-	-			123 ± 0.15	-			
	III	5 ± 0.2	-	-	-			44 ± 0.17	-			
	IV	29 ± 0.4	-	-	-			200 ± 0.26	-			

**Figure 8:**
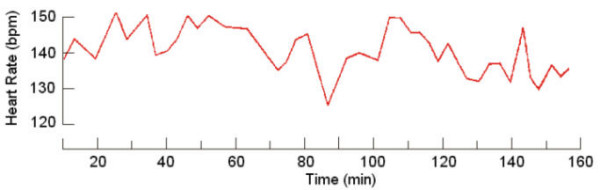
Fetal cardiotachogram for "active" period

**Figure 9:**
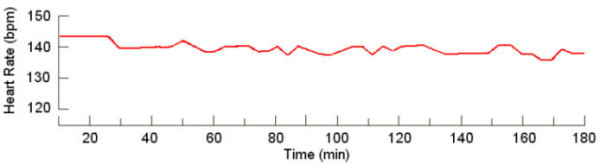
Fetal cardiotachogram for "quiet" period

Our investigation demonstrated that concentration of type I oscillations on a cardiotachogram during "quiet" hours was four times lower than in "active" hours; concentration of type II and IV oscillations was twice as low in "quiet" hours as compared to "active" periods. The "flat" (III) type oscillations were four times as prevalent during "quiet" hours as during "active" hours. Statistical processing of the findings regarding the duration of each type of the oscillations and "active" and "quiet" hours of the fetus yielded a reliable result (p < 0.001) (Fig. 8, Fig. 9).

**Figure 10:**
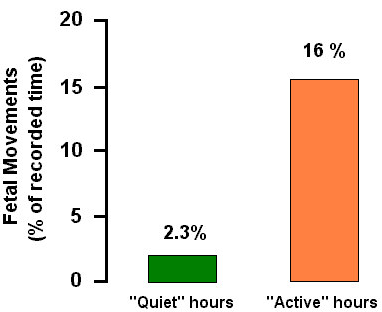
Fetal locomotor activity in "quiet" and "active" hours (36 – 40 weeks gestation)

The analysis of locomotor activity during the defined periods of rest and activation of the fetus showed that in "quiet" hours the recording of fetal movements lasted for 11 min and 5 sec, which corresponded to 2,3% of the recording time. In "active" hours, fetal locomotor activity augmented by 7–8 times and was equal to 91 min and 10 sec, which corresponded to 16% of the recording time (Fig. 10).

Apart from the analyses mentioned, we thought it reasonable to calculate the number of fetal heart contractions with the values lower than 120 bpm and higher than 150 bpm, which were encountered in hourly portions of daily cardiotachograms. The data obtained pointed to a significant prevalence of the frequencies lower than 120 bpm in "quiet" hours, whereas frequencies higher than 150 bpm were either solitary or were not registered at all.

ECGs recorded from the pregnant women were also analyzed. It was found that during nocturnal sleep the duration of "flat" type cardiotachograms exceeded significantly that of types I, II, and IV. In the day-time, during mother's waking period, "flat" type cardiotachograms were either absent or occasionally appeared on hourly recordings within a short space of time (2–5 min).

Our investigation has demonstrated that "active" periods of the fetus are characterized by the elevation of the levels of physiological functions, which is expressed by the predominance of "peak-like", "rounded" and "mixed" oscillations with high levels of intra-minute fluctuation and variability of HR, as well as by the prevalence of frequency values higher than 150 bpm and enhanced locomotor activity of the fetus. "Quiet" hours show typical reduction of HR variability, predominance of "flat" (type III) oscillations, significant prevalence of frequencies lower than 120 bpm, and sharply decreased locomotor activity [2,4,8,9,18,29].

This evidence suggests that the fetus develops a sleep-like state from 4 a.m. to 9 a.m. and from 2 p.m. to 7 p.m. The duration of night-time sleep in the healthy pregnant women was 8 h 10 min. The EEG analysis showed that common structural characteristics of sleep together with each of its phases are typical of those of healthy individuals being in the relative resting state [6].

The analysis of fetal locomotor activity during night sleep of the pregnant women revealed 1051 ± 2.6 fetal movements, observed from 10 p.m. to 4.30 a.m. (sleep cycle I – 207 ± 1.4 movements, II – 315 ± 2.3, III – 11 ± 1.4, IV – 508 ± 2.1). At the same time, from 4.30 a.m. to 8 a.m. there were only 136 ± 1.2 movements (sleep cycle V – 196 ± 1.1, VI – 40 ± 1.6). During "active" hours (7 p.m-4 a.m.), irrespective of the mother's sleep phase, the fetus was observed to be active, as inferred from the increased number of its movements. On the other hand, locomotor activity of the fetus was 7–8 fold decreased in "quiet" hours (from 4 a.m. to 9 a.m.).

**Figure 11:**
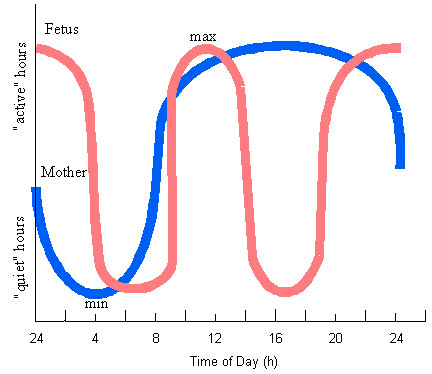
Correlation of quiet and active periods for mother

Thus, locomotor activity of the fetus did not affect the course of nocturnal sleep and its cyclicity. Consequently, as in adults, acceleration and deceleration of physiological activity take place in a healthy full-term fetus. The curve depicting changes in the levels of fetal physiological functions bears a biphasic character; the levels are reduced in the morning (4 a.m. to 9 a.m.) and in the afternoon (2 p.m. to 7 p.m.) and increased during the day-time (9 a.m. to 2 p.m.) and evening-night (7 p.m. to 4 a.m.) periods (Fig. 11).

According to many authors, changes in the majority of physiological processes in humans (body temperature, activity of the cardiovascular system, respiration rate, etc.) manifest themselves in constant elevations of the levels from 8 a.m. to 1 p.m., with a slight decrease between 1 p.m. and 2 p.m., and continued elevation reaching maximal values by 4 p.m. to 6 p.m. The second minimal value of the parameters is observed at 2 to 3 a.m. The daily rhythm oscillation of blood adrenalin and adrenohypophyseal system activity fluctuate within the range of an opposite phase, reaching peak values at 6 a.m. to 9 a.m. These data suggest that the periods from 4 a.m. to 9 a.m. and from 7 p.m. to 1 a.m. are transitional stages during which the mother's physiology shifts from one functional level to another, which is a natural functional load for both the mother and the fetus. It is possible to suggest that the process involved in the intrauterine development of the fetus requires the availability of a relatively persistent homeostasis; the fetus employing its intrinsic adaptive capacities of responding to the rhythmic changes in the levels of maternal physiological functions becomes active when these levels are reduced and decreases its own physiological activity when they are elevated. The data obtained has led us to the conviction that the levels of functioning of the fetal physiological systems do comply with the state of maternal organism but run with a reverse phase.

### Stage 3

Issues regarding the onset and formation of the fetal intrauterine rhythm were studied on 28 pregnant women at the gestational term of 16–28 weeks (at 16–20 weeks – 8 pregnant participants, at 21–24 weeks – 12, at 26–28 weeks – 8). The self-designed methodological approach previously employed in our investigations was entirely preserved. Analyses of daily rhythmograms were performed using the original method described above [7,17,19]. The results showed regular rhythms of daily fluctuations of HR in all 28 pregnant women. Sleep duration in this group was 8 hrs 32 min. The general structural characteristics and each phase of sleep were typical of those of a healthy individual.

The analysis of 24-hour CTGs of the fetus demonstrated a clear-cut daily rhythm of heart rate and locomotor activity in 21 fetuses (16–20 weeks gestation – 6 cases; 21 – 24 weeks gestation – 8 cases; 26–28 weeks gestation – 7 cases).

Just as in a full-term fetus at 16–28 weeks gestation, the fetus' background cardiotachogram for "active" hours, from 9 a.m. to 2 p.m. and from 7 p.m. to 4 a.m., was represented by mixed (type IV) oscillations with markedly expressed "peak-like" and "rounded" types. During "quiet" periods (4 a.m. to 9 a.m. and 2 p.m to 7 p.m.), the background rhythmogram was represented by "flat" (type III) oscillations. The locomotor activity in "active" hours was 11 (in a full-term fetus 6–7) times as intensive as compared to that in "quiet" periods of the day. Consequently, as early as at 16–20 weeks pregnancy the fetus clearly expressed 24-hour rhythms of the heartbeat and locomotor activity. On the other hand, in 7 cases (out of 28) the analysis of daily cardiotachograms showed mostly "mixed" oscillations during both "active" and "quiet" periods with "peak-like, "rounded" and "flat" types (types I, II, III) being observed with different intensity against the background cardiotachograms. In these 7 cases (at 16–20 weeks' gestation – 2 cases; 21–24 weeks' gestation – 4 cases; 26–28 weeks' gestation – 1 case), the concentration of "flat" (type) oscillations in "active" hours was equal to 17 ± 0.6 min, i.e. 3.03% of the total recording time from 9 a.m. to 2 p.m. and from 7 p.m. to 4 a.m. In "quiet" hours the concentration of flat oscillations was 14 ± 1.03 min, corresponding to 28% of the total recording time from 4 a.m. to 9 a.m. and from 2 p.m. to 7 p.m. The locomotor activity of these 7 fetuses was 3.5 fold higher in "active" hours than that in "quiet" periods. Comparative analysis of these findings for 21 fetuses at 16–28 weeks' gestation with expressed fetal rhythms showed that the concentration of "flat" (type III) oscillations in "active" hours made up 49 ± 0.6 min, i.e. 8.7% of the total recording time. During "quiet" hours, the concentration of "flat" oscillations was 56.2% of the total recording time. Fetal movements in "active" periods were observed to be 11 times more than in "quiet" hours.

The study allows assuming that, at the gestational age of 16–20 weeks, the hypothalamo-hypophyseal system of a healthy fetus reaches the degree of maturation which is sufficient to provide well-developed capacities for adaptation to the environment. A healthy fetus at the gestational age of 16–20 weeks has pronounced daily rhythms of the heartbeat and locomotor activity. The fetuses in which we failed identify any distinctly expressed rhythms of heart beat and locomotor activity were included in the "risk" group. They were given a repeated observation following 3–4 weeks. Absence of clear-cut rhythmicity at 20–24 weeks gestation indicates developmental retardation.

The periods that we classified as "active" are characterized by the predominance of "peak-like" and "rounded" types of cardiotachograms. These oscillations appear against the background of "mixed" types, which occupy the major portion of the 24-hour period. "Quiet" hours are characterized by the prevalence of "flat" cardiotachograms. We have determined "active" (9 a.m. to 2 p.m. and 7 p.m. to 4 a.m.) and "quiet" (4 a.m. to 9 a.m. and 2 p.m. to 7 p.m.) periods for the fetus.

### Stage 2

According to literature data [31-38], there are three types of fetal response to functional testing: "acceleration", "deceleration" and "zero-type" reaction. The best response to functional load is "acceleration". "Deceleration" points to a decrease in compensatory mechanisms, while "zero-type" reaction is indicative of an unsatisfactory condition of the fetus.

The analysis of 24-hour fetal CTGs showed that in "active" hours the background cardiotachograms were represented by mixed-type (IV) oscillations within the range of 126.0 ± 3.4 bpm to 148.6 ± 3.8 bpm; the intra-minute fluctuations equaled 7.5 ± 2.0 bpm. At functional loading, an "acceleration" type response was observed. The baseline rhythm following the loading was 136.8 ± 1,3 bpm. Response time to loading made up 32.0 ± 1.1 sec [1,3,5,8,30].

Of the 2,500 fetuses investigated, FHR accelerations were seen in 2,004 (80.2%) of cases at fetal movements. The remaining 496 fetuses showed deceleration and "zero-type" oscillations induced by locomotor activity. During "quiet" hours (2 p.m. to 7 p.m.), the fetal background CTG's were represented by "flat" (type III) oscillations. FHR fluctuations were observed within the range of 131.6 ± 3.1 bpm with the average of 136.2 ± 2.9 bpm. The baseline rhythm was 138.2 ± 2.6 bpm. The intra-minute fluctuations were 1.9 ± 0.8 bpm to 4.5 ± 0.7 bpm with the average of 3.2 ± 0.7 bpm. Out of the 2,500 recordings of fetal external ECGs in "quiet" hours, fetal movements were recorded in 37 cases. No changes in FHR were observed in these cases. At functional loading in "quiet" hours the fetuses revealed "zero-type" response, i.e. there was no reaction at all. The baseline rhythm level following the loading was 137.6 ± 1.7 bpm.

Evaluation of the 2,500 fetuses by Apgar Scale identified 2,257 cases (90.7%) with 8 to 10 points and 133 cases (9.3%) with 7 to 8 points.

**Figure 12:**
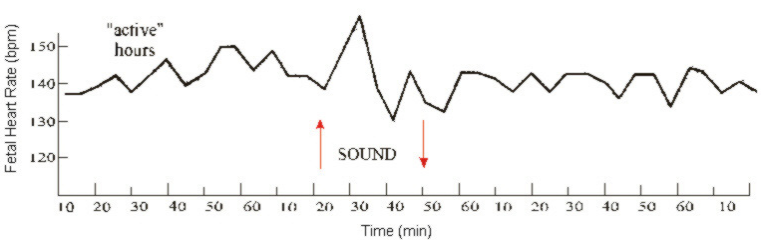
Response of the fetus to functional load in "active" hours ("acceleration" type)

**Figure 13:**
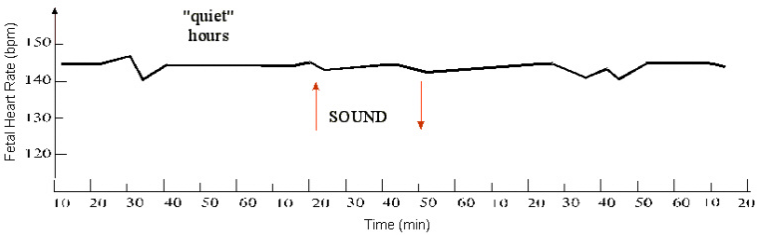
Response of the fetus to functional load in "quiet" hours ("zero" type)

In summary, our investigation has clearly showed that in "active" hours a fetus with efficient compensatory-adaptive mechanisms responds to functional loads by HR acceleration (Fig. 12). No reaction is observed in "quiet" periods. However, the "zero"-type fetal reaction recorded by us within the period from 2 p.m. to 9 p.m. does not indicate unsatisfactory condition of the fetus but rather is suggestive of a definite reduction of functional levels of the fetal physiological systems, which is necessary for vital activity. Although conventionally recognized as an indicator of poor state of the fetus, this type only calls for precise attention when recorded in fetal "active" hours (Fig. 13).

## Conclusion

Although we found no significant daily rhythmicity in heart rate per se in the human fetus, we developed a new method for the assessment of 24-hur fetal cardiotachogram that allowed us to identify daily rhythmicity in the short-term pattern of heart beating. The analysis of four types of oscillation – designated as "peak-like", "rounded", "flat," and "mixed" – revealed that "acceleration" and "deceleration" in the physiological functions of the fetus occurs in a way similar to that of an adult. "Active" hours (9 a.m. to 2 p.m. and 7 p.m. to 4 a.m.) and "quiet" hours (4 a.m. to 9 a.m. and 2 p.m. to 7 p.m.) were determined for the fetus. Fetal locomotor activity did not influence the course and cyclicity of the mother's nocturnal sleep.

It can be assumed that the fetus, during the intrauterine development requires the availability of a relatively persistent homeostasis; the fetus employing its intrinsic adaptive capacities of responding to the rhythmical changes in the levels of maternal physiological functions becomes active when these levels are reduced and decreases its own physiological activity when they are elevated. A healthy fetus at the gestational age of 16–20 weeks has pronounced daily rhythms of the heartbeat and locomotor activity. Absence of clear-cut rhythms at 20–24 weeks gestation indicates developmental retardation.

We showed that a fetus with efficient compensatory-adaptive mechanisms responds to functional loading by the heart beat acceleration in "active" hours. No reaction is observed in "quite" periods. However the "zero"-type fetal reaction recorded does not point to unsatisfactory condition of the fetus, but rather is suggestive of a definite reduction of functional levels of the fetal physiological systems which is necessary for vital activity. The "zero" type, conventionally recognized as an indicator of poor state of the fetus, should be taken into consideration merely in "active" fetal hours.

The results of this study provide empirical bases for the assessment of the intrauterine state of the fetus, thus advancing the prognosis for both pregnancy and labor. The investigations have laid down the foundation for fetal chronobiology and chronotherapy, having established the principle of interdependence and conformity between maternal and fetal biological clock.

## Competing interests

The author(s) declare that they have no competing interests.

## Authors' contributions

**Kintraia P **– Supervisor of the study. Performed data analysis.

**Zarnadze M **– Chief experimenter. Collected material for stages 1, 2, and 3. Conducted data analysis. Wrote the article.

**Kintraia N **– Collected material for stages 2 and 3. Conducted data analysis.

**Kashakashvili I **– Collected material for stage 3.

## List of abbreviations

bpm – beats per minute

CTG – cardio tachogram

ECG – electrocardiogram

EEG – electroencephalogram

FHR – fetal heart rate

h – hour(s)

HR – heart rate

NST – non-stress test

sec – second(s)
